# High-dependency units play a key role in the treatment of a Chinese military patient who developed liver failure while abroad

**DOI:** 10.1186/s40779-019-0220-y

**Published:** 2019-09-16

**Authors:** Chen Li, Hai-Bin Su, Xiao-Yan Liu, Li-Na Zhang, Jin-Hua Hu

**Affiliations:** Liver Failure Treatment and Research Center, The Fifth Medical Center of Chinese PLA General Hospital, Beijing, 100039 China

**Keywords:** High-dependency units, Liver failure, Treatment, Military patient

## Abstract

High-dependency units (HDUs) provide high-level care to patients who suffer from single organ failure, with the exception of respiratory failure requiring mechanical ventilation; HDUs serve as an intermediary between general wards and Intensive Care Units. Due to military and civilian needs, our hospital has established a unique HDU for patients with liver disease in China. A Chinese military officer in the United Nations Peacekeeping Forces in South Sudan was transferred to our HDU for liver failure treatment in 2018. The patient’s disease status, nutrition, sleep habits, and psychological behaviour were monitored on different scales. The patient was provided with vascular monitoring, telemetry, pulse oximetry, drug treatment, nutritional support, sleep intervention, psychological intervention, and humanistic care by a multidisciplinary treatment team. After treatment, the patient recovered and avoided liver transplantation. Based on the experience with this HDU, this new model may create an efficient treatment process for military and civilian patients with severe liver disease at home or abroad.

High-dependency units (HDUs), also known as intermediate care units and step-down units, were established to provide a level of care between the general ward and the Intensive Care Unit (ICU) [1–3]. In the United Kingdom, this level of care is thought to be suitable for patients suffering from single organ failure, with the exception of those with respiratory failure requiring mechanical ventilation [1–3]. However, the indications for HDU admission vary regionally and even within institutions [1–4]. HDUs are believed to confer advantages in the management of postoperative, paediatric, cardiac, and respiratory syndromes, and HDU admission may reduce the need for unnecessary ICU admission [4–6]. As such, it has been argued that HDUs may be cost-saving and improve access to the ICU for patients who are truly dependent on critical care resources [6]. Furthermore, HDUs may facilitate earlier ICU discharge and reduce the likelihood of ICU readmission [7]. One study even suggested that HDUs may improve patient satisfaction [8]. Last, increased humanistic care in HDUs may help reduce the incidence of post-intensive care syndrome in patients who are otherwise at risk [9]. However, the role of HDUs in the management of patients with severe liver disease (SLD) has received limited attention [10]. The purpose of this letter is to describe the first HDU for SLD in China and showcase its performance using an example case.

## Description of our liver disease HDU

In China, liver disease affects approximately 300 million people, which accounts for a significant proportion of the global burden [11, 12]. Patients with SLD, such as acute liver failure, acute-on-chronic liver failure and acute decompensation in the setting of cirrhosis, are frequently admitted to intensive care. In China, these patients often present with coagulopathy, renal insufficiency, and altered mental status, but not respiratory failure [13]. Many SLD patients could therefore be managed in HDUs with close monitoring.

In recent years, the Chinese military has observed a high incidence of liver disease among military service personnel overseas [14–16]. Frequently, medical resources overseas cannot meet the treatment needs of these patients. For these reasons, a specialized HDU for patients with SLD was established in our hospital. The 7 bed unit was opened in 2017. For context, our hospital has 1200 beds and approximately 50,000 inpatient visits per year.

Admission to this specialized HDU requires the presence of SLD with serious complications, including ascites, acute kidney injury, hepatic encephalopathy (≤ grade 2), gastrointestinal bleeding, infection, and sepsis. Patients with respiratory failure and high-grade hepatic encephalopathy (≥grade 3) are admitted to the ICU. Patients can be admitted from any of the following locations: emergency department, the general ward when deteriorating, and the ICU after stabilization. An additional function of the HDU is to stabilize, monitor, and manage patients prior to liver transplantation. In normal times, the HDU is used to treat domestic military and civilian patients with SLD. However, priority is given to military personnel with SLD who have been overseas.

The HDU nurse-to-patient ratio is 1:2—1:3. Clinical staff, including physicians and nurses, are present 24 h/d, 7 d/week. The staff is composed of a multidisciplinary treatment team consisting of doctors, nurses, clinical pharmacists, dieticians, physiotherapists, and psychologists. All team members evaluate the condition of the patient and work together to develop an individualized treatment plan for the patient. The chief physician of the HDU is the team leader and is responsible for coordinating weekly meetings to discuss each patient’s condition and management plan. Specific services and monitoring available to patients in the unit include arterial catheterization for invasive monitoring of blood pressure, central venous catheterization, telemetry, and continuous pulse oximetry. Admitted patients also receive an assessment of their nutritional status with recommendations for optimization, a psychological evaluation and management as necessary, and interventions to optimize sleep. In addition, the HDU can provide bedside artificial liver support (ALS) and renal replacement therapy. Moreover, the unit has a telemedicine terminal that is used to coordinate medical evacuations and provide management expertise for military service members with SLD prior to HDU admission.

## Case report of an overseas military patient treatment

**Fig. 1:**
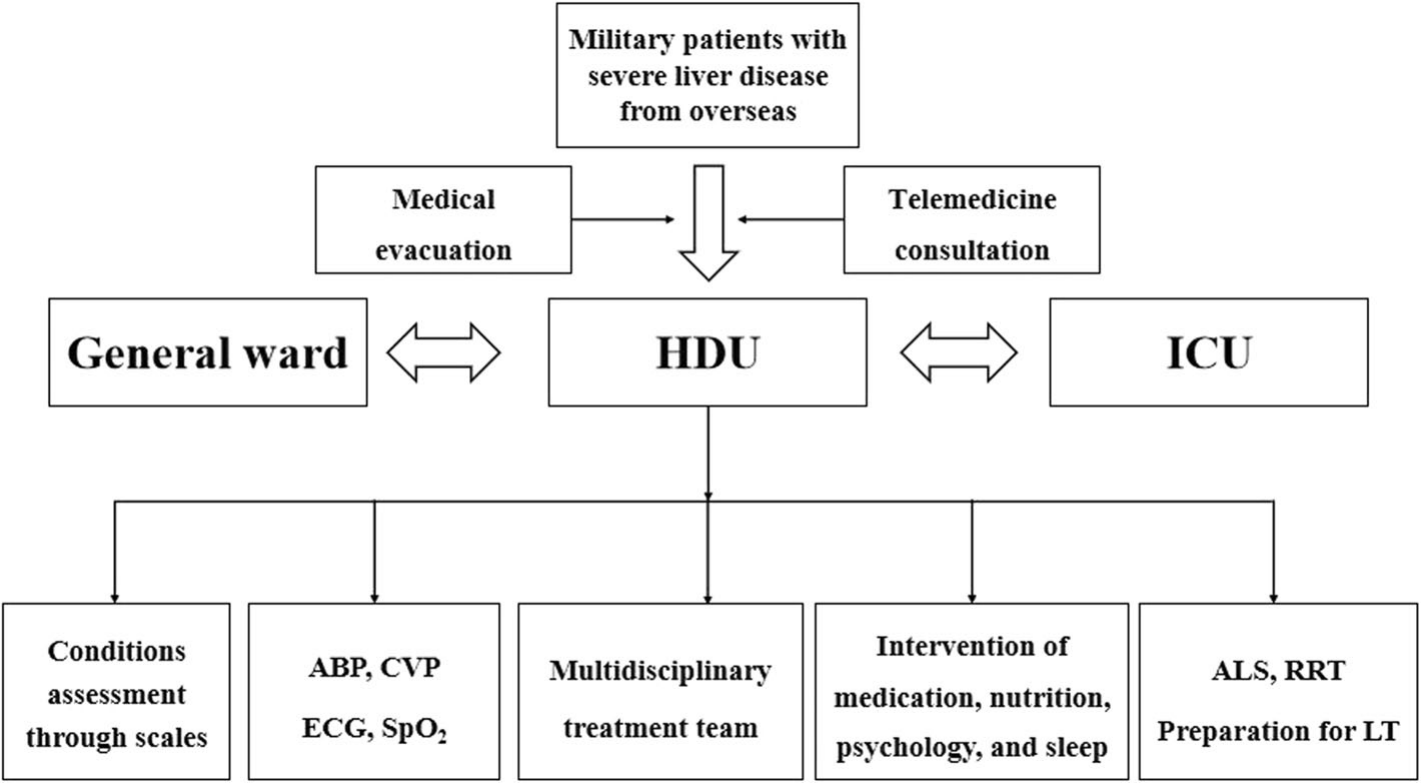
Treatment process for military patients with severe liver disease (SLD) from overseas. When military patients experience SLD overseas, they can be transferred to our high-dependency units (HDUs) for treatment by telemedicine consultation and medical evacuation. In this HDU, patients can receive therapy from a multidisciplinary treatment team. Arterial blood pressure (ABP) monitoring, central venous pressure (CVP) monitoring, electrocardiogram (ECG), and pulse oxygen saturation (SpO_2_) monitoring are provided for the patients. Artificial liver support (ALS), renal replacement therapy (RRT), preparation for liver transplantation (LT), nutritional support treatment, psychological assessment, and sleep interventions are provided to the patients. According to their condition, patients are selected for transfer to the general ward or the intensive care unit (ICU)

Our specialized HDU has established a bridge between the general ward and the ICU (Fig. 1). It was recently tested in the treatment of an overseas military patient with liver failure. This Chinese officer in the United Nations Peacekeeping Forces of South Sudan was transferred to our HDU with liver failure in February 2018. This 41-year-old man, with a past history notable for hypertension and diabetes, had no prior history of liver disease. In South Sudan, he developed fever, fatigue, anorexia, and jaundice. On laboratory evaluation, he was noted to be coagulopathic with the following additional abnormalities: alanine aminotransferase (5410 U/L; normal < 40 U/L), aspartate aminotransferase (3475 U/L; normal < 40 U/L), total bilirubin (225.1 μmol/L; normal 3.4–20.5 μmol/L), prothrombin activity (21.5%; 65–130%) and international normalized ratio (2.12; normal 0.8–1.2). Following the HDU-based telemedicine consultation, a preliminary diagnosis of acute viral hepatitis was suggested, and the patient was evacuated by air to our HDU.

Upon admission, vascular monitoring (arterial and central venous line), telemetry, and pulse oximetry were initiated. He was prepared for ALS and liver transplantation. Additional testing confirmed a diagnosis of hepatitis A virus-related liver failure with nutritional risk, sleep disorders, and psychological disorders. In the setting of our multidisciplinary care plan, the patient recovered and avoided liver transplantation. This case exemplifies what has become an efficient HDU-based strategy in the management of patients with SLD. The characterization of his overall condition upon admission to the HDU, specific assessments and interventions, and measures at HDU discharge on day 31 are characterized in Table 1.

**Table 1:** Interventions, scale scores at HDU admission and discharge of the patient

Variable	Admission score	Intervention	Discharge score
SOFA	4	Magnesium isoglycyrrhizinate, ursodeoxycholic acid, adenosylmethionine butanesulfonat, clotting factor	2
MELD	23.4		5.4
NRS 2002	4	Carbohydrate, amino acid, fat, vitamins, dietary fibre	1
PSQI	21	Sedative, physiotherapy	10
SCL90-R	188	Psychological counselling, humanistic care	108
GSI	2.1		1.2
PST	40		14
PSDI	3.5		2.3
SOM	2.2		1.2
O-C	2.2		1.0
IS	1.2		1.1
DEP	2.5		1.2
ANX	2.0		1.1
HOS	2.2		1.0
PHOB	1.7		1.1
PAR	1.5		1.0
PSY	1.8		1.2

ANX. Anxiety; *DEP* Depression, *GSI* General symptomatic index, *HDU* High-dependency unit, *HOS* Anger-hostility, *I-S* Interpersonal sensitivity, *MELD* Model for end-stage liver disease, *NRS* Nutrition risk screening; *O-C* Obsessive-compulsive, *PAR* Paranoid ideation, *PHOB* Phobic anxiety, *PSDI* Positive symptom distress index, *PSQI* Pittsburgh sleep quality index, *PST* Positive symptom total, *PSY* Psychoticism, *SCL90-R* Symptom check list 90-revised, *SOFA* Sequential organ failure assessment, *SOM* Somatization

## Conclusions

This specialized HDU allows a proportional use of medical resources and likely prevents ICU admissions for patients like the one we have described. Our specialty unit and multidisciplinary treatment team optimize the diagnosis, management, evaluation, and triage of patients with SLD. The efficiency of this model is very likely to save the lives of military and civilian patients with SLD, a condition that may progress rapidly to death without appropriate expertise and resources. Consideration should be given to replicating our model at other military or civilian medical centres, including mobile centres, military medical ships and aircrafts.

## Data Availability

Not applicable.

## Abbreviations

ALS: Artificial liver support
ANX: Anxiety
DEP: Depression
GSI: General symptomatic index
HDU: High-dependency unit
HOS: Anger-hostility
ICU: Intensive care unit
I-S: Interpersonal sensitivity
MELD: Model for end-stage liver disease
NRS: Nutrition risk screening
O-C: Obsessive-compulsive
PAR: Paranoid ideation
PHOB: Phobic anxiety
PSDI: Positive symptom distress index
PSQI: Pittsburgh sleep quality index
PST: Positive symptom total
PSY: Psychoticism
SCL90-R: Symptom check list 90-revised
SLD: Severe liver disease
SOFA: Sequential organ failure assessment
SOM: Somatization
